# Feasibility of Vis-NIR spectroscopy approach to predict soil biological attributes in arid land soils

**DOI:** 10.1371/journal.pone.0311122

**Published:** 2024-09-25

**Authors:** Elias Hosseini, Mehdi Zarei, Ali Akbar Moosavi, Reza Ghasemi-Fasaei, Majid Baghernejad, Hasan Mozaffari

**Affiliations:** 1 Department of Soil Science, College of Agriculture, Shiraz University, Shiraz, Iran; 2 Department of Agriculture and Natural Resources, Higher Education Center of Eghlid, Eghlid, Iran; Government College University Faisalabad, Pakistan, PAKISTAN

## Abstract

Visible and near-infrared (Vis-NIR) reflectance spectroscopy has recently emerged as an efficient and cost-effective tool for monitoring soil parameters and provides an extensive array of measurements swiftly. This study sought to predict fundamental biological attributes of calcareous soils using spectral reflectance data in the Vis-NIR range through the application of partial least square regression (PLSR) and stepwise multiple linear regression (SMLR) techniques. The objective was to derive spectrotransfer functions (STFs) to predict selected soil biological attributes. A total of 97 composite samples were collected from three distinct agricultural land uses, i.e., sugarcane, wheat, and date palm, in the Khuzestan Province, Iran. The samples were analyzed using both standard laboratory analysis and proximal sensing approach within the Vis-NIR range (400–2500 nm). Biological status was evaluated by determining soil enzyme activities linked to nutrient cycling including acid phosphatase (ACP), alkaline phosphatase (ALP), dehydrogenase (DEH), soil microbial respiration (SMR), microbial biomass phosphorus (P_mic_), and microbial biomass carbon (C_mic_). The results indicated that the developed PLSR models exhibited superior predictive performance in most biological parameters compared to the STFs, although the differences were not significant. Specifically, the STFs acceptably accurately predicted ACP, ALP, DEH, SMR, P_mic_, and C_mic_ with R^2^_val_ (val = validation dataset) values of 0.68, 0.67, 0.65, 0.65, 0.76, and 0.72, respectively. These findings confirm the potential of Vis-NIR spectroscopy and the effectiveness of the associated STFs as a rapid and reliable technique for assessing biological soil quality. Overall, in the context of predicting soil properties using spectroscopy-based approaches, emphasis must be placed on developing straightforward, easily deployable, and pragmatic STFs.

## Introduction

The Intergovernmental Technical Group on Soils (ITPS), formed under the auspices of the Food and Agriculture Organization’s (FAO) Global Soil Partnership, has freshly outlined soil health as ’the capacity to uphold the productivity, diversity, and environmental benefits of terrestrial ecosystems’ [1]. The vitality of soil health cannot be overstated, as it enables the sustenance of life by facilitating the cycling of organic matter (OM) and nutrients through the involvement of plants and soil organisms [2]. Soil health, influenced by various factors such as soil physicochemical properties, nutrient availability, soil health indicator enzymes, and microbial diversity, is fundamental to agricultural sustainability [3]. Soil enzymes, as crucial organic components in soil, act as biological indicators of soil fertility due to their biocatalytic properties and protein content [4]. Additionally, the composition and abundance of soil microorganisms significantly affect the absorption and transformation of nutrients crucial for soil-crop interaction. Consequently, soil enzyme activity (EA) and microbial biomass (C_mic_) serve as vital indicators of soil health [5]. However, traditional laboratory-based analyses of EA and C_mic_ are both time-consuming and involves the use of various chemicals. Considering this, leveraging visible and near-infrared (Vis-NIR) spectroscopy emerges as a promising alternative for estimating enzyme activities, leveraging distinctive spectral effects associated with different enzymes. It’s important to note, however, that the efficacy of "Vis-NIR" spectroscopy in estimating enzyme activities remains a topic of discussion [6]. The application of "Vis-NIR" spectroscopy, a segment of the electromagnetic spectrum, is gaining traction as a precision agriculture approach for assessing soil physicochemical attributes both in the field and laboratory settings [7,8]. This non-invasive analytical technique proves cost-efficient, delivers swift outcomes, and has the capability to deduce various components from a singular spectrum. Furthermore, it eliminates the need for chemical substances in the analysis process, thereby posing no harm to the environment [9,10]. The utilization of NIR spectroscopy in agriculture and food science gained traction during the 1980s, and its application in soil science saw significant growth in the late 1990s [11]. This method enables the prediction of diverse soil characteristics through direct engagement with particular absorbance/reflectance bands or by establishing an indirect correlation with soil properties that exhibit a clear connection to soil spectra [12]. However, the analysis of effective single-bands for estimating various soil attributes using conventional methods is promising as a result of spectral multi-collinearity [13].

The "Vis-NIR" spectroscopy has emerged as a promising approach for determining various soil properties, covering physical and hydraulic attributes like particle size, aggregation, and hydraulic conductivity [10,14]; chemical components like soil organic carbon (SOC), cation exchange capacity (CEC), various macro- and micro-nutrients, and pH [13,15–17]; and biological factors like C_mic_, microbial respiration, EA, and microbial groups [18–20]. Several studies have demonstrated the effectiveness of "Vis-NIR" spectroscopy for predicting different soil biological factors. For example, Zornoza et al. [21] successfully utilized Vis-NIR spectroscopy to predict soil biological properties, like enzyme activities, with high accuracies. Similarly, Dick et al. [22] reported high determination coefficient (R^2^) values for various soil properties, showcasing the potential of spectroscopy in Vis-NIR region for measuring various soil properties with a single spectral scan. In terms of predicting enzyme activities, Reeves et al. [23] proposed potential explanations for the NIR results, pointing towards indirect correlations with C or N, emphasizing the potential association with biologically active N. Soriano-Disla et al. [24] highlighted the strong correlations between biological attributes and the quantity and quality of soil organic matter (SOM), underscoring the success of "Vis-NIR" in estimating biological properties. Moreover, specific signature wavelengths might also play a role in these estimations.

Different approaches such as partial least square regression (PLSR), support vector machines (SVM), fuzzy rule-based models, and artificial neural networks (ANNs) have been employed to analyze spectral data effectively [25–29]. Yet, certain methods exhibit a "black box" nature, signifying a dearth of straightforward, user-friendly models in the current studies for estimating soil attributes based on spectral data [30,31]. Performing "PLSR" (as a straightforward approach) to analyze spectral data can help identify effective bands for predicting various soil properties and develop spectrotransfer functions (STFs). It is noteworthy that estimating soil attributes with "STFs" is simpler, more practical, user-friendly, and does not necessitate complex computer software compared to PLSR models [13].

Beyond the previously mentioned limitation, there is a scarcity of studies in the existing literature that focus on creating specific and user-friendly STFs for the prediction of various soil properties. In this regard few studies developed specific STFs to predict basic physicochemical properties [13], soil bacterial abundance and diversity [32], soil hydraulic properties [13,33], erodibility factor [25,34], and SOM [35]. This gap underscores the critical need to develop tailored STFs for easily predicting soil biological properties, ultimately saving time, costs, and reducing the use of chemicals.

Given the dominance of sugarcane, wheat, and date palm crops in Khuzestan Province, Iran, this study aimed to estimate biological properties, as important indicators of soil health, using the PLSR method and develop STFs for the calcareous soils of the mentioned land uses. Our objectives were to assess the potential of "Vis-NIR" spectral reflectance data for predicting significant biological properties, such as soil microbial respiration (SMR), microbial carbon (C_mic_), microbial phosphorus (P_mic_), and activities of enzymes like acid phosphatase (ACP), alkaline phosphatase (ALP), and dehydrogenase (DEH) using the PLSR method. Moreover, our objective is to formulate STFs utilizing impactful "Vis-NIR" spectral reflectance bands (SRB) for predicting the aforementioned soil biological properties through stepwise multiple linear regression (SMLR). In addition, we seek to compare the predictive capabilities of the "PLSR" method and "SMLR-STFs" for predicting these soil biological properties. Lastly, we aim to investigate the correlation between certain fundamental soil physio-chemical properties with the measured soil biological properties.

## Materials and methods

### Study area and sample collection

The area of study was an agricultural center in Khuzestan Province that is located in the southwest of Iran. The study area is located between the latitudes of 48° 27′-48° 31′ E and 31° 03′-31° 10′ N. The elevation spans from 16 to 18 m above the mean sea level. Although the Province’s climate varies greatly, the majority of its regions and urban areas are arid terrain, and the average annual precipitation is 266 mm [36]. The moisture and temperature regime are aridic and hyperthermic, respectively. The soil in the study area varies in calcium carbonate equivalent (CCE) values, ranging from 42.2 to 55%, and consists predominantly of soluble dolomite and calcite limestone (Table 1). The soil samples of study area are primarily categorized as Inceptisols according to Soil Taxonomy [37].

**Table 1:** Statistics summarizing and fitting parameters for the normal distribution of the selected soil attributes.

Attribute^†^	Unit	N	Min	Max	Mean	SD	CV (%)	VS	SK	KR	KS^††^
ACP	μg PNP g^-1^ h^-1^	97	35.2	164	90.2	29.8	33	Moderate	0.536	-0.308	0.097^ns^
ALP	μg PNP g^-1^ h^-1^	97	62	385	235	68.69	29.1	Moderate	-0.471	0.013	0.093^ns^
DEH	μg TPF g^-1^ h^-16^	97	2	122	20.9	19	90.8	High	2.16	7.79	0.161*
ln(DEH)	ln(μg TPF g^-1^ h^-16^)	97	0.693	4.47	2.62	0.921	35.1	High	-0.266	-0.845	0.088^ns^
SMR	mg CO_2_ kg^-1^ h^-24^	97	1.98	5.67	3.88	0.941	24.2	Moderate	-0.186	-0.832	0.102^ns^
P_mic_	μg P g^-1^	97	4.16	14	8.34	2.35	28.2	Moderate	0.454	-0.526	0.103^ns^
C_mic_	μg C g^-1^	97	142	423	263	62.2	23.6	Moderate	0.454	-0.163	0.076^ns^
SOM	%	97	0.681	2.38	1.22	0.365	29.8	Moderate	1.07	1.24	0.130^ns^
CCE	%	97	42.3	55	47.5	1.59	3.35	Low	0.733	4.72	0.114^ns^
Sand	%	97	19.2	54.6	38.3	7.44	19.4	Moderate	0.068	-0.267	0.075^ns^
Clay	%	97	3.33	24.12	14.3	5.45	37.9	High	-0.258	-0.803	0.083^ns^
Silt	%	97	32.4	58.5	47.3	5.82	12.3	Low	-0.583	-0.043	0.126^ns^
pH	-	97	7.14	7.89	7.54	0.201	2.66	Low	-0.129	-0.108	0.132^ns^
EC	dS m^-1^	97	1.09	25	4.82	4.37	90.6	High	2.03	5.24	0.198**
ln(EC)	ln (dS m^-1^)	97	0.091	3.22	1.24	0.804	64.9	High	0.356	-0.948	0.134^ns^
MWD	mm	97	1.30	3.24	1.90	0.33	17.5	Moderate	0.88	1.03	0.098^ns^

†: ACP: Acid phosphatase activity; ALP: Alkaline phosphatase activity; ln(DEH): Natural logarithms (ln) of dehydrogenase activity; SMR: Soil microbial respiration; P_mic:_ Microbial biomass phosphorus; C_mic_: Microbial biomass carbon; SOM: Soil organic matter; CCE: Calcium carbonate equivalent (lime); Sand: Sand content; Clay: Clay content; Silt: Silt content; pH: pH of saturated paste; EC: Electrical conductivity of saturated extract; MWD: Mean weight diameter.

††: N, Min, Max, Mean, SD, CV, VC, SK, KR and KS are the number of soil samples, minimum, maximum, mean, standard deviation, coefficient of variations, variability classes, skewness coefficient, kurtosis coefficient, and the statistics of Kolmogorov-Smirnov normality test, respectively. ns means lack of significant difference with the normal distribution

** and * show significant differences with the normal distribution (departure from normal distribution) at the probability levels of 1 and 5%, respectively (which became normal with the logarithmic transformation).

**Fig 1:**
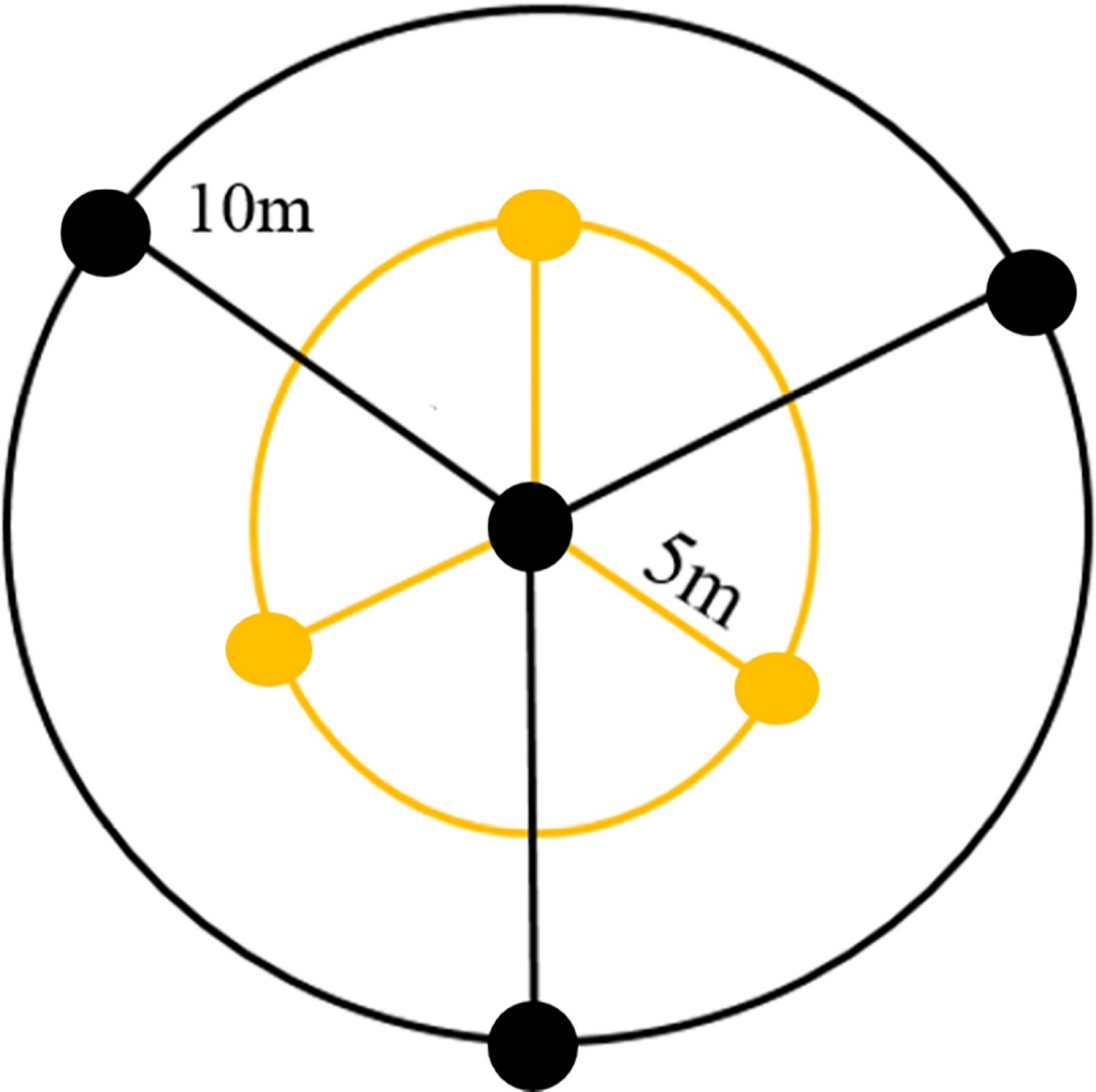


Ninety-seven soil samples were gathered from the 0–30 cm depth across various land uses, encompassing cultivated fields of wheat, date, and sugarcane [38]. The samples were collected from a square sampling grid of 75 m distance interval similar to the method used by Vâgen et al. [39], and were taken to the laboratory for further analysis. Individual samples were composite samples including seven subsamples collected from a circular sampling scheme of 20 m diameter (Fig 1). All field sampling processes were conducted with prior arrangement with landlords.

### Soil analysis

The samples underwent sieving using a 2 mm mesh and were subsequently transported to a laboratory at the Department of Soil Science, Shiraz University, Shiraz, Iran, for the evaluation of specific biological characteristics. A part of the samples was air-dried and subjected to standard methods for evaluating their physicochemical attributes. The analysis of soil texture, encompassing sand, silt, and clay contents, followed the USDA classification system [40,41]. This involved a combination of wet-sieving and sedimentation methods, as outlined by Gee and Bauder [42]. Additionally, the pH of the saturated paste was gauged using a glass electrode pH-meter [43]. Electrical conductivity (EC), of the saturated extract was measured using an EC-meter following the method proposed by Rhoades [44]. The calcium carbonate equivalent (CCE) and SOM were measured through back titration with HCl [45] and the Walkley-Black wet combustion method [46], respectively.

The mean weight diameter of aggregates (MWD) was measured using dry-sieving method [47]. For this aim, 50 g of air-dried and passed through an 8 mm sieve soil was placed on a series of sieves with hole diameters of 4, 2, 1, 0.8, 0.6, 0.4, 0.2 and 0.075 mm [48], which were sorted based on the size of the holes from top to bottom along with a tray. The sieves were vibrated during 5 min with a vibration height of 1 mm and an acceleration of 5 times more than the gravity acceleration.

The activities of enzymes including ACP (EC 3.1.3.2) and ALP (EC 3.1.3.1) of each sample was detected employing a method introduced by Tabatabai [49] and the results were indicated as μg p-nitrophenol (pNP) g^−1^h^−1^. In the case of the controls, the substrate was introduced into the mixture once the reaction had concluded.

The DEH was assessed using a method outlined by Tabatabai [49]. This technique involved the reduction of Triphenyltetrazolium chloride (TTC) to produce a red compound known as triphenylformazon (TPF). The activity level was assessed by monitoring the absorbance at 485 nm using a UV-Visible spectrophotometer.

To estimate soil C_mic_, the direct extraction method devised by Vance et al. [50] was employed. This approach employed 0.5 M K_2_SO_4_ as an extractant and chloroform for fumigant. The carbon content attributable to chloroform-labile carbon was subsequently calculated by establishing the difference in carbon content between the chloroform-fumigated and non-fumigated subsamples [K_EC_-factor = 0.37 (converts the organic C-flush to microbial C)]. Phosphate levels in the soil microbial biomass were determined by introducing CHCl to the soil to break down microbial cells. The content of microbial-P (P_mic_) released to 0.5 M NaHCO_3_ extracts (pH 8.5) was measured. The calculations for total microbial-P were based on the difference in P_mic_ extracted by NaHCO_3_ from CHCl_3_-treated and untreated samples [K_EP_-factor = 0.4 (assuming that 40% of P in the biomass is rendered extractable as inorganic P by chloroform)]., as per the methodology by Brookes et al. [51].

For soil microbial respiration (SMR) measurement, soil samples were incubated in sealed containers at 25°C. The CO_2_ emitted by microorganisms was captured using NaOH and quantified through titration with HCL [52]. The Kolmogorov-Smirnov test was performed to check the normality of the studied using SPSS Statistics 26 software package.

### Vis-NIR spectroscopy

Approximately 25 g of soil samples, which had been air-dried, sieved using a 2 mm sieve, and entirely mixed, were carefully placed in glass containers. The spectral absorbance of these soil samples was monitored by a spectrophotometer approach (Rapid Content Analyzer, "NIRS XDS", Metrohm Company, Switzerland) across 4200 wavelengths within the "Vis-NIR" (400–2500 nm). The absorbance data were recorded at wavelength intervals of 0.5 nm, with a wavelength accuracy of less than 0.05 nm and a bandpass of 8.75 nm [13,16]. To simplify the analysis, the spectra were treated as independent variables (IV), considering a 1 nm increment between data points.

To obtain mean absorbance spectrum, three scans were conducted, each approximately three minutes apart. To ensure data accuracy and remove unwanted noise, the spectral absorbances obtained in the ranges of 400 to 417 nm and 2483 to 2500 nm were excluded [13]. The collected absorbance spectral data (A) were then converted to reflectance (R) values. To enhance the spectral data quality and improve predictions by removing turbulence, pre-processing techniques including Savitzky-Golay derivation [53] with smoothing points of 5, as well as a second-order polynomial approximation [54] and the standard normal variate, SNV approach were employed utilizing the Unscrambler X v. 9.7 software package (CAMO, Technologies Inc., Massachusetts, USA). This comprehensive approach helped optimize the spectral data for further analysis and interpretations.

### PLSR

The current study aimed to develop regression models to predict soil biological properties using spectral reflectance bands (SRB) in the "Vis-NIR" region. The dependent variables (Y) were the soil biological properties, while the independent variables (X) were the SRB. To build the regression models, we randomly divided the entire dataset into two subsets namely (i) a calibration dataset (75%) and (ii) a validation dataset (25%). The calibration dataset was used to train and optimize the PLSR models in the hidden layer. Subsequently, these algorithms were validated using the validation datasets. To identify the most effective spectral reflectance (ESR) bands for predicting the studied soil attributes, the plots of regression coefficient (B) values vs. wavelengths resulted from the "PLSR" analyzed using Unscrambler X v. 9.7 software. The presence of peaks in these plots indicated potential positive/negative correlations between SRB at specific wavelengths and studied properties. The reflectance values at these peak wavelengths were selected as important single-bands and incorporated into SMLR models. Notably, only some of the crucial single-bands (but not all) were included in the final "STFs" [25,35]. All aspects of the PLSR analysis, including model development, identification of important bands from the B plots, and developing STFs for predicting the studied soil attributes, were conducted through the processed reflectance spectra.

### Developing STFs

The process of deriving STFs involved employing the stepwise multiple linear regression (SMLR) approach. This method sought to establish STFs based on the highest level of correlation observed between the relevant soil properties and the ESR of individual bands. It is crucial to mention that the input IV in the STFs developed were the ESR bands identified through analysis of PLSR. For this study, the forward SMLR approach was employed. In such approach, variables were added based on the level of tolerance significance determined by the F-test probability of 0.05.

The calibration dataset employed for analysis of PLSR was also used for the development of the STFs. Subsequently, the models that were developed underwent testing using the validation dataset, which coincided with the one utilized for PLSR analysis.

### Model evaluation

In evaluating the model’s performance, various goodness of fit criteria, which included some metrics such as the determination coefficient (R^2^), the ratio of performance to interquartile range (RPIQ), normalized root means square error (NRMSE), and Nash-Sutcliffe coefficient (NS) were employed as described by Mozaffari et al. [48] Notably, the RPIQ statistic is particularly useful for assessing non-normal variables, as highlighted by Briedis et al. [55]. The RPIQ value ranges from 1.5 to 3 was considered for an acceptable model as suggested by Jia et al. [56].

The assessment of accuracy was according to R^2^ values, and five levels of accuracy were taken into account. An R^2^ value falling within the range of 0.90 to 1 was classified as excellent, while very good accuracy ranged from 0.75 to 0.90. Models with an R^2^ value between 0.65 and 0.75 were deemed good, and those with R^2^ values ranging from 0.50 to 0.65 were considered acceptable, moderate, or fair. Finally, models with R^2^ values of less than 0.50 were labeled as poor [13]. The NRMSE was considered as a metric for quantifying the error between predicted and observed values, with a range from zero (indicating the highest accuracy) to infinity. To facilitate classification, NRMSE (%) values falling within 0–10, 10–20, 20–30, and >30 are considered as excellent, good, fair, and poor, respectively, as outlined by Bannayan and Hoogenboom [57].

As for the NS statistic, it gauges model efficiency and can vary from one (representing the highest accuracy) to negative infinity. Ritter and Muñoz-Carpena [58] along with Feng et al. [59] have established that NS values falling within 0.9–1, 0.8–0.9, 0.65–0.8, and <0.65 are categorized as very good, good, acceptable, and unsatisfactory, respectively.

To accomplish curves fitting and calculate the aforementioned statistics, the Excel software package (version 2013) was used.

## Results

### Statistical summaries and correlation among the studied properties

Table 1 displays an overview of the statistical summaries and normality assessment for the analyzed soil attributes. Within the physicochemical attributes, the minimum and maximum coefficient of variations (CV) were observed for pH (2.66%) and EC (90.6%), respectively. Similarly, for the biological properties, the minimum and maximum CV were associated with C_mic_ (23.1%) and DEH (90.8%), respectively. As per Wilding (1985), CCE, silt, and pH fall within the low variability category (0 < CV ≤ 15%); P_mic_, C_mic_, SMR, ALP, ln (DEH), SOM, and sand fall within the moderate variability category (15 < CV ≤ 35%); ACP, DEH, clay, EC, and ln (EC) belong to the high variability category (CV > 35%). Previous studies have reported CV values ranging from 4.54 to 393% for physicochemical properties [13] and 17.1 to 42.8% for biological properties [22] of soils. Moreover, Rezaee et al. [60] documented CV values ranging from 2 to 56% for the physicochemical characteristics of paddy soils in their study. The Kolmogorov-Smirnov normality test indicated that DEH and EC did not adhere to a normal distribution. Therefore, the logarithmic transformation was applied to normalize the mentioned attributes (Table 1).

Additionally, Table 2 indicates the Pearson correlation coefficients (r) of the investigated attributes. It is evident that P_mic_, ACP, ALP, and ln (DEH) exhibit significant positive correlations (*p* < 0.05 and 0.01) with clay (ranging from 0.23 to 0.54) and significant negative correlations (*p* < 0.05 and 0.01) with EC (ranging from -0.25 to -0.45). Furthermore, SOM demonstrated a significant positive correlation with P_mic_ and C_mic_, with r values of 0.24 (*p* < 0.05) and 0.32 (*p* < 0.01), respectively. Conversely, C_mic_, SMR, ACP, and ln (DEH) displayed negative correlations (r > 0.3) with pH significantly (*p* < 0.01).

**Table 2:** Pearson correlation coefficients (r) between the studied soil properties.

	**ACP**														
**ACP**	1^††^	**ALP**													
**ALP**	0.48**	1	**ln(DEH)**												1
**ln(DEH)**	0.34**	0.35**	1	**SMR**											
**SMR**	0.04	0.04	0.32**	1	**P**_**mic**_										
**P**_**mic**_	0.21	0.19	0.40**	0.13	1	**C**_**mic**_									
**C**_**mic**_	-0.10	0.29**	0.21	0.35**	0.25*	1	**SOM**								
**SOM**	-0.06	-0.06	0.09	0.19	0.32**	0.24*	1	**CCE**							0
**CCE**	-0.01	-0.01	-0.09	0.02	-0.36**	-0.14	-0.07	1	**Sand**						
**Sand**	-0.05	-0.05	-0.19	-0.18	-0.26*	-0.11	-0.27*	0.03	1	**Clay**					
**Clay**	0.23*	0.23*	0.45**	0.05	0.24*	0.01	0.02	-0.09	-0.66**	1	**Silt**				
**Silt**	-0.16	-0.16	-0.19	0.19	0.11	0.14	0.33**	0.05	-0.67**	-0.12	1	**pH**			
**pH**	-0.33**	-0.33**	-0.30**	-0.32**	-0.11	-0.46**	0.14	-0.09	-0.09	0.04	0.09	1	**ln(EC)**		-1
**ln(EC)**	-0.20	-0.20	-0.31**	0.26*	-0.26*	0.25*	0.09	-0.01	0.31**	-0.64**	0.22*	-0.41**	1	**MWD**	
**MWD**	0.26*	0.07	0.35**	-0.09	-0.03	0.13	0.27*	0.03	-0.07	-0.02	0.11	-0.25*	0.12	1	

†: ACP: Acid phosphatase activity, ALP: Alkaline phosphatase activity, ln(DEH): Natural logarithms (ln) of dehydrogenase activity, SMR: Soil microbial respiration, P_mic:_ Microbial biomass phosphorus, C_mic_: Microbial biomass carbon, SOM: Soil organic matter; CCE: Calcium carbonate equivalent (lime), Sand: Sand content, Clay: Clay content, Silt: Silt content, pH: pH of saturated paste, ln(EC): Natural logarithms (ln) of (electrical conductivity of saturated extract), MWD: Mean weight diameter.

††: * and ** demonstrate the significant correlation between properties in probability levels of 5 and 1%, respectively.

### Analysis of PLSR to predict soil biological attributes

#### ACP, ALP, and DEH

**Fig 2:**
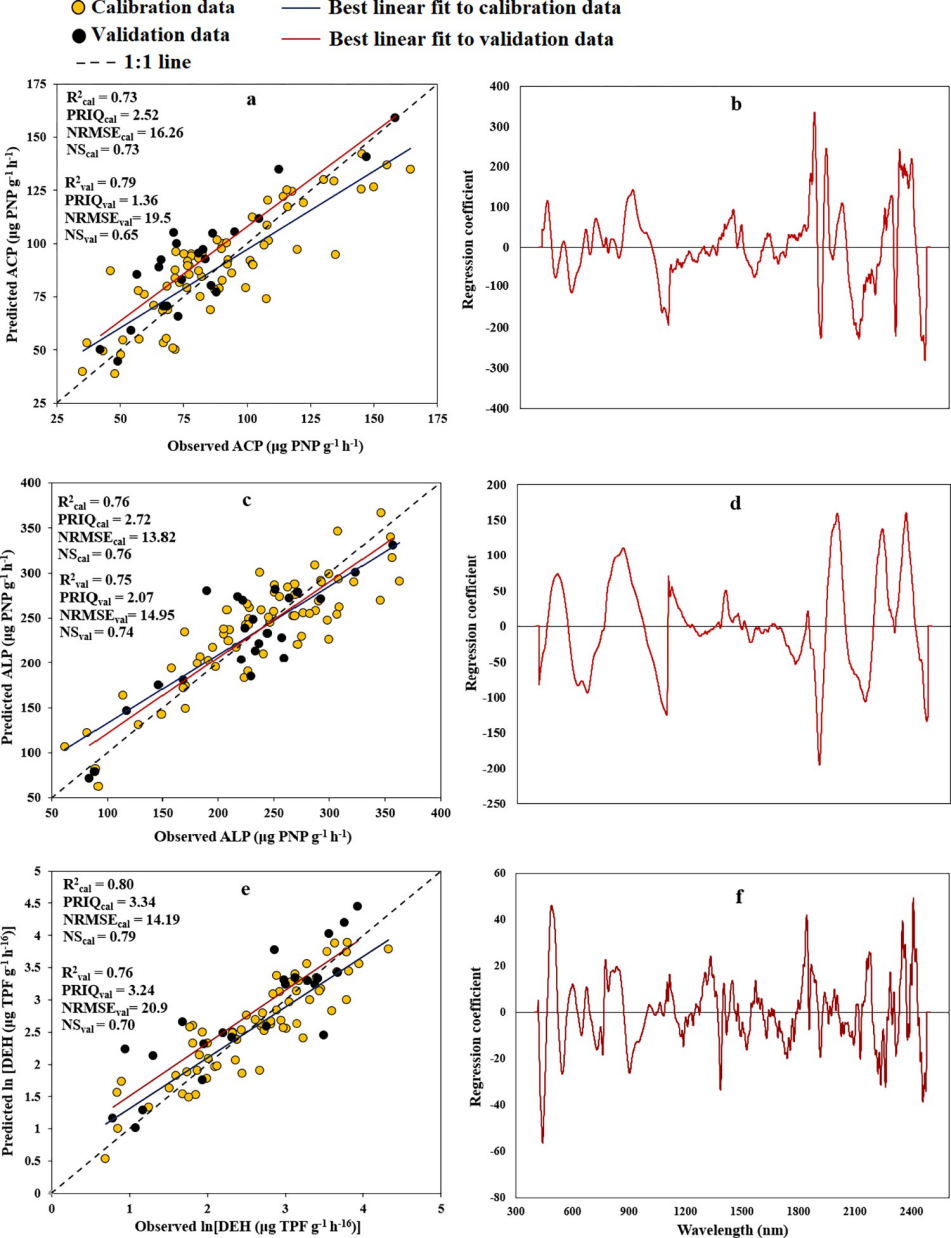


Fig 2 shows the observed values versus Vis-NIR spectra-predicted ACP, ALP, and DEH using accompanied by their corresponding plots of regression coefficients (B) against wavelengths based on PLSR analysis. The ACP, ALP, and DEH prediction was conducted with R^2^_val_ values of 0.79, 0.75, and 0.76; NRMSE_val_ of 19.5, 14, and 20.9%; NS_val_ of 0.65, 0.74, and 0.70; and RPIQ_val_ of 1.36, 2.07, and 3.34, respectively.

#### SMR, P_mic_ and C_mic_

**Fig 3:**
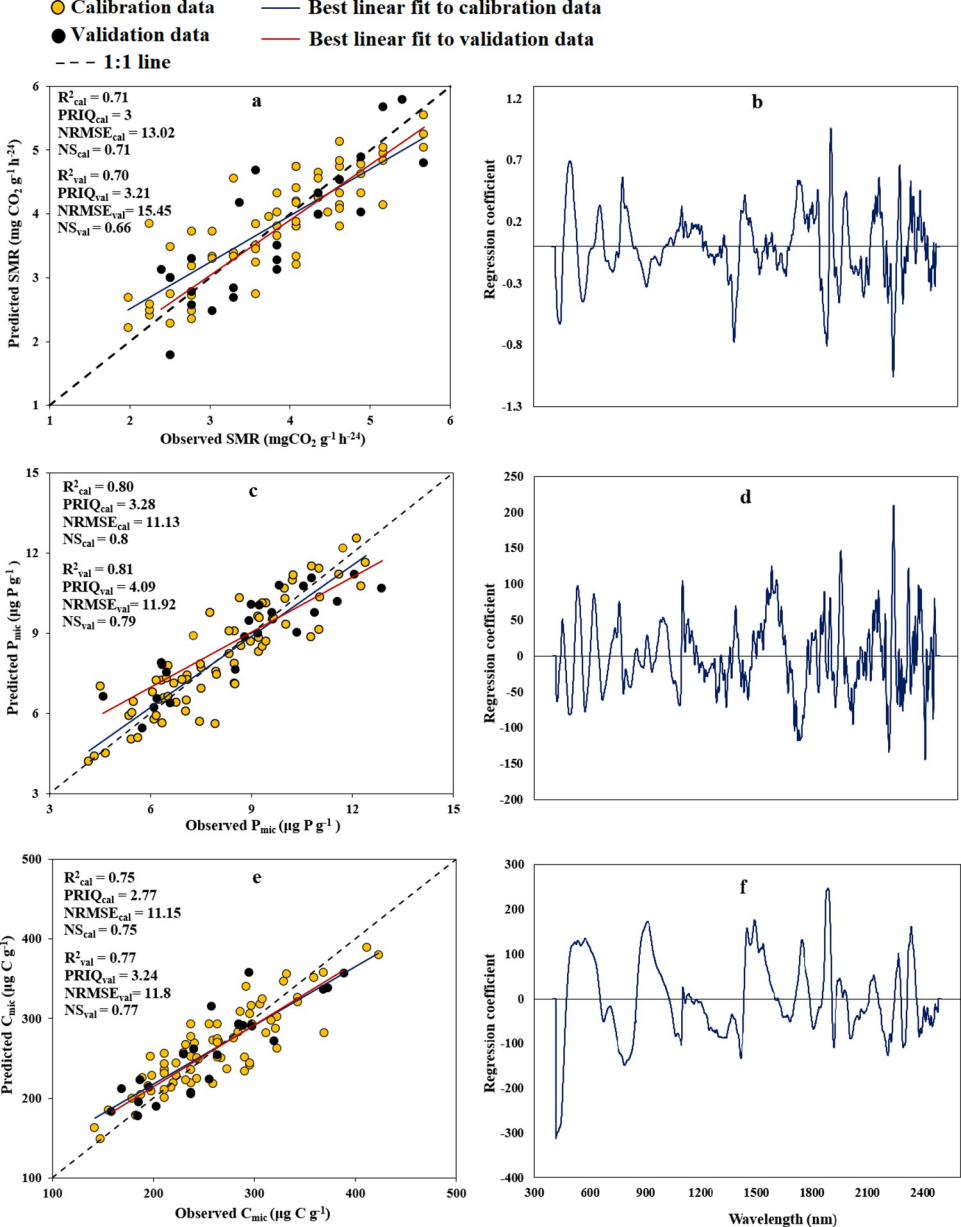


Fig 3 depicts the capability of the PLSR technique in forecasting SMR, P_mic_, and C_mic_ attributes through the utilization of Vis-NIR SRB. Additionally, the goodness of fit criteria and graphs illustrating the relationship between B values and wavelengths are presented. The PLSR method predicted SMR, P_mic_, and C_mic_ with R^2^_val_ of 0.70, 0.81, and 0.77; NRMSE_val_ of 15.4, 11.9, and 11.8%; NS_val_ of 0.66, 0.79, and 0.77; and RPIQ_val_ of 3.21, 4.09, and 3.24, respectively.

### Development of STFs to predict soil biological attributes

#### ACP, ALP, and DEH

The B values (as shown in Fig 2), derived from analysis of PLSR, revealed that numerous influential and significant SRB were predominantly within wavelength ranges of 418–1104 nm, 1384–1407 nm, and 1891–2482 nm. These bands were instrumental in predicting soil enzyme activity components (ACP, ALP, and DEH) and in formulating STFs. Utilizing the identified influential bands from the analysis of PLSR, we formulated the subsequent STFs (Eqs 1 to 3). These equations, employing 8 SRB and the SMLR approach, were developed for predicting ACP, ALP, and DEH, respectively.


$$\begin{array}{l} ACP\;(\mu g\;PNP\;g^{-1}\;h^{-1})=228.1-9497.6(R_{655})-55(R_{2032})+3068.1(R_{418})\\ \;+23856.6(R_{896})-26265.4(\;R_{1096})+3718.8(R_{2347})\;\begin{array}{c} i=18\\ m=8 \end{array}\\ \;+10534.8(R_{1384})-4986(R_{2108}) \end{array}$$ (1)

$$\begin{array}{l} ALP(\mu g\;PNP\;g^{-1}\;h^{-1})=733.1-15975.9(R_{672})+8693.9(R_{2482})-13035.4(R_{1904})\\ \;+5794.4(R_{419})+26412.8(R_{863})-47567.8(R_{1095})\;\begin{array}{c} i=15\\ m=8 \end{array}\\ \;+25965.9(R_{1104})+10078.8(R_{1407}) \end{array}$$ (2)

$$\begin{array}{l} DEH\;(\mu g\;TPF\;g^{-1}\;h^{-16})=12.3-41.7(R_{2475})+197(R_{2169})+321(R_{1891})\\ \;-332(R_{545})-1000(R_{441})+1133(R_{489})\;\begin{array}{c} i=13\\ m=8 \end{array}\\ \;-484(R_{2079})+125(R_{601}) \end{array}$$ (3)

**Fig 4:**
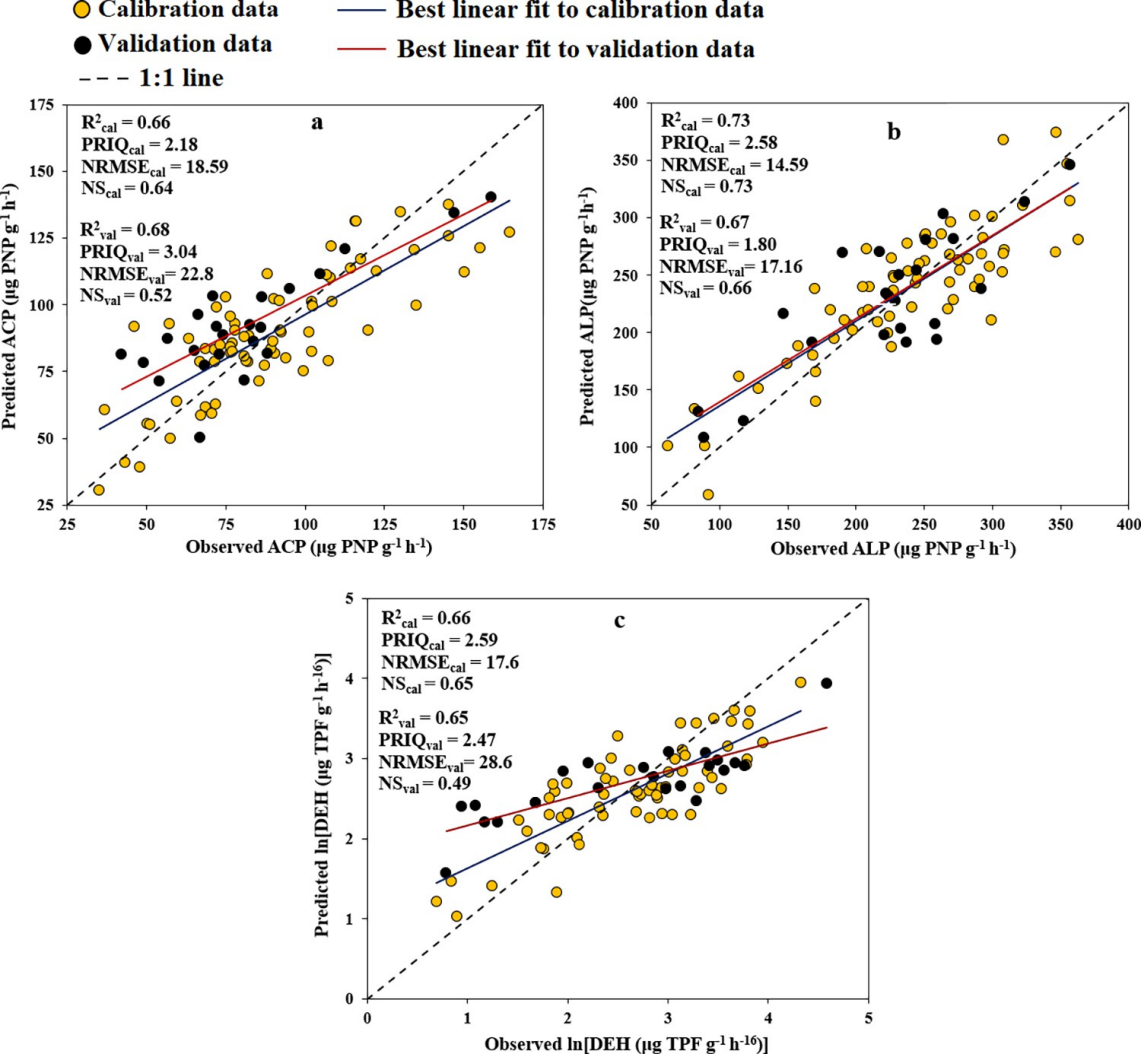


In the given context, Rs represents the spectral reflectance value at wavelength s, i signifies the number of selected important single-bands from the PLSR analysis (B plot) incorporated into the SMLR model, and m denotes the number of effective single-bands present in the developed STF. The efficacy of the STFs in predicting ACP, ALP, and DEH is illustrated in Fig 4. The R^2^_val_ values for ACP, ALP, and DEH were 0.68, 0.67, and 0.65, respectively. The NRMSE_val_ values were 22.8%, 17.1%, and 28.6%, NS_val_ values were 0.52, 0.66, and 0.49, and RPIQ_val_ values were 3.04, 1.8, and 2.47, respectively.

#### SMR, P_mic_ and C_mic_

In Fig 3, it can be observed that the important and effective bands for predicting SMR, P_mic_, and C_mic_ cover a wide range of the spectrum (418–2391 nm). Using these ESR bands based on the B values and SMLR approach, we developed the following STFs (Eqs 4 to 6) to predict SMR, P_mic_, and C_mic_. It was revealed that 8, 11, and 7 SRB were observed in the STFs for prediction SMR, P_mic_, and C_mic_, respectively.


$$\begin{array}{l} SMR(mg\;CO_{2}\;g^{-1}\;h^{-24})=0.2786+1.20(R_{913})+21.6(R_{1743})+1.03(R_{731})\\ \;+18.2(R_{2345})-26.8(R_{2249})+9.83(R_{1995})\;\begin{array}{c} i=15\\ m=8 \end{array}\\ \;-13.3(R_{1387})–11(R_{2154}) \end{array}$$ (4)

$$\begin{array}{l} P_{mic}(\mu g\;P\;g^{-1})=33.9+63.6(R_{424})+2441(R_{490})-2666(R_{453})+78.7(R_{1935})\\ \;+432(R_{2332})-399(R_{2387})-74.8(R_{623})+595(R_{1135})\;\begin{array}{c} i=19\\ m=11 \end{array}\\ \;–1080(R_{1089})-499(R_{2030})+854(R_{1394}) \end{array}$$ (5)

$$\begin{array}{l} C_{mic}(\mu g\;C\;g^{-1})=408+9483(R_{1682})+37423(R_{1447})-28300(R_{418})\;\begin{array}{c} i=18\\ m=7 \end{array}\\ \;+20712(R_{533})-19644(R_{2391})-37141(R_{1336})+9414(R_{2337}) \end{array}$$ (6)

**Fig 5:**
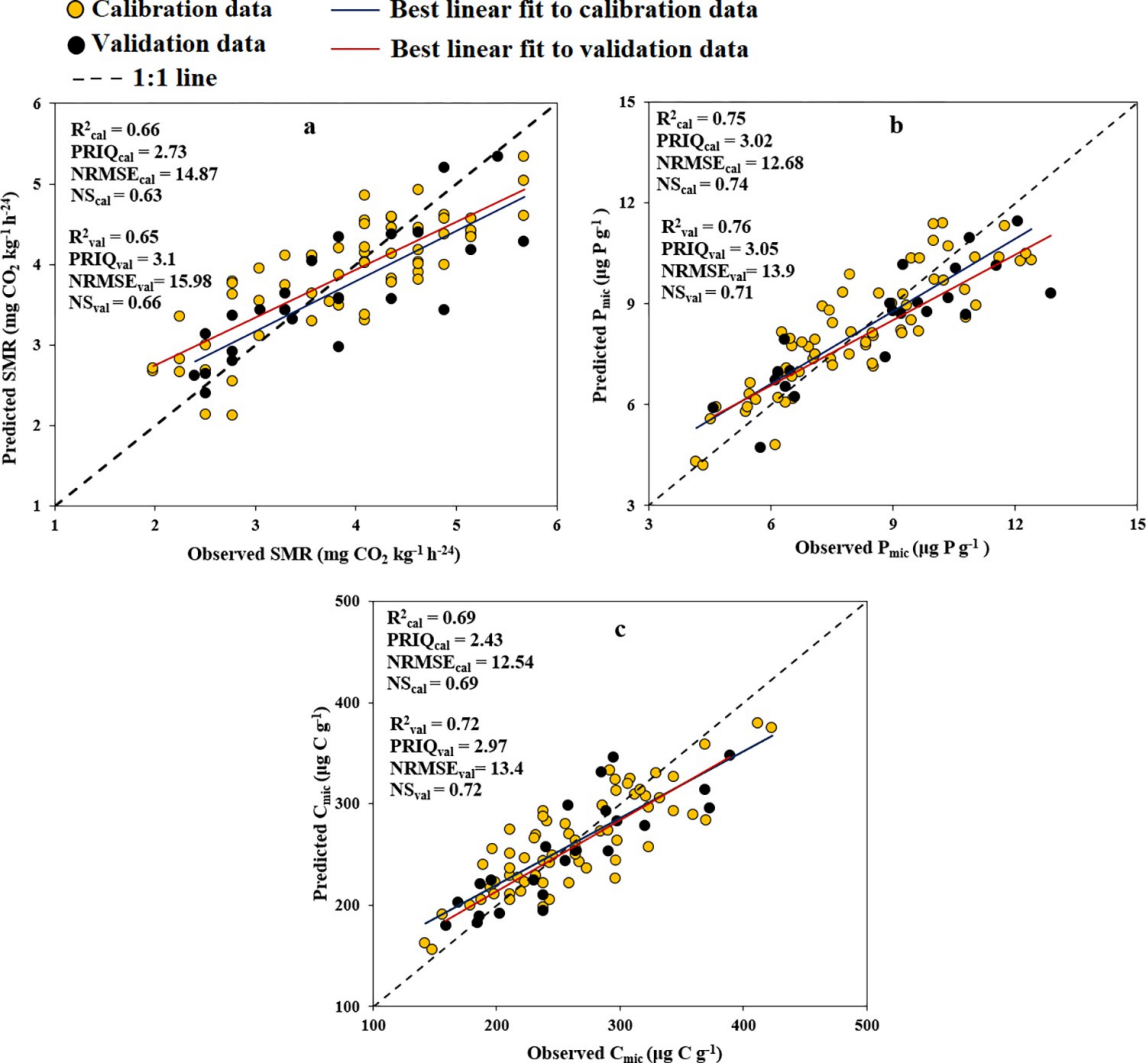


Fig 5 displays scatter plots comparing obtained values to predicted values, accompanied by goodness-of-fit criteria for forecasting SMR, P_mic_, and C_mic_ using STFs. The associated STFs predicted these properties with R^2^_val_ values of 0.65, 0.76, and 0.72; NRMSE_val_ of 15.9, 13.9, and 13.4%; NS_val_ of 0.66, 0.71, and 0.72; and RPIQ_val_ of 3.1, 3.5, and 2.94, respectively.

### Assessing the efficacy of the PLSR method and STFs in predicting soil biological attributes

**Fig 6:**
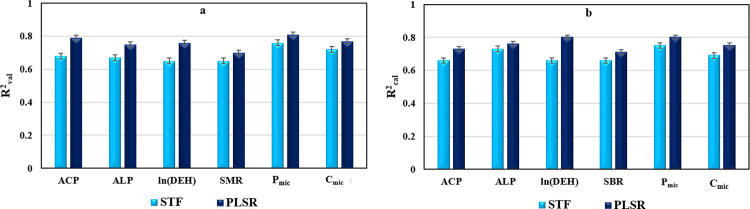


**Fig 7:**
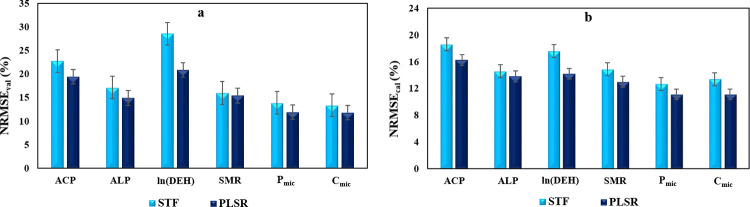


Figs 6 and 7 provide a comparative analysis of the R^2^_val_ and NRMSE_val_ values for the "PLSR" models and "STFs" (Eqs 1 to 6), utilizing "Vis-NIR" reflectance spectra to predict soil biological attributes. The highest R^2^_val_ value (0.81) was noted for P_mic_ prediction using PLSR, while the lowest R^2^_val_ value (0.65) was observed for ln(DEH) and SMR prediction using STF. Additionally, Fig 7 illustrates that the highest NEMSE_val_ values (20.9% and 28.6%) were recorded for ln(DEH) prediction using PLSR and STFs, respectively. Conversely, the lowest NEMSE_val_ values (11.92% and 11.8%) were attained for P_mic_ and C_mic_ prediction using PLSR.

## Discussion

### Correlation between selected soil physico-chemical and biological attributes

In the present paper, as indicated in Table 2, we observed a positive correlation between clay content and microbiological parameters, specifically extracellular enzymes activity. The observed correlation is likely a result of the evolution of complexes involving clay-phosphodiesterase and/or clay-organic matter-phosphodiesterase, as suggested by Babaeian et al. [33] these complexes play a role in safeguarding the enzyme, possibly by preserving the conformation of the active site through adsorption onto both internal and external surfaces of smectite and/or organic matter [33].

Furthermore, negative correlations obtained between EC and pH with enzymes activity. The impact of pH on the stability of soil enzymes holds significant importance, given that exposure to extreme pH values can irreversibly deactivate enzymes crucial to nutrient (N, C, P, and S) transformations and humus formation. Changes in enzymes activity within soils were linked to variations in hydrogen ion concentration due to the reversible reaction of ionizing or deionizing prototrophic groups in the enzyme-protein’s active center and irreversible denaturation of the enzyme [61].

Salinity has the potential to impede the activity of various soil enzymes, such as "dehydrogenase", "β-glucosidase", "urease", "protease", ACP and ALP, "arylsulfatase", and "argininamide hydrolase" [62]. In saline soils, salts alter root exudate patterns, to which soil microorganisms adapt for survival under high salt concentrations. This adaptation requires energy expenditure to preserve osmotic balance, resulting in a reduction in microbial population and root attachment capability in the rhizosphere [63].

Moreover, a positive correlation was observed between SOM and P_mic_. Additionally, a negative correlation was obtained between pH with C_mic_ and SMR. Various parameters such as temperature, pH, moisture content, and SOM content influence soil organism activity [64,65]. These parameters likely play a role in providing nutrients for microbial communities. In addition, it can be seen in Table 2 that the ACP and ln(DEH) have a positive and significant correlation with MWD. In a research, Bharti et al. [66] investigated the effect of three aggregate fractions (large macroaggregates, small macroaggregates, and micro-aggregates) on the activity of soil enzymes. They stated that aggregate fractions of small macroaggregates (250–2000 μm) exhibited higher specific enzyme activities (except DEH), when expressed in terms of SOC. It is likely that the disintegration of soil aggregates and increased enzyme activity in large macroaggregates contributed to the liberation of enzymes that were previously bound and immobilized [66]. In line with findings from earlier research, there is a significant and favorable association between enzyme activity and MWD [67].

### Prediction of soil biological attributes

#### ACP, ALP, and DEH

In accordance with the NRMSE classification guidelines, ACP (utilizing PLSR) and ALP (utilizing both PLSR and STF) demonstrated accurate predictions (NRMSE_val_ within the range of 10–20), while ACP (using STF) and DEH (using both PLSR and STF) displayed moderately accurate predictions (NRMSE_val_ falling within the range of 20–30) based on Vis-NIR spectra. Additionally, following the NS categorization, Vis-NIR spectroscopy yielded acceptable accuracy (NS_val_ of 0.65–0.80) in predicting ACP (using PLSR), ALP (using both PLSR and STF), and DEH (using PLSR model). However, it resulted in unsatisfactory accuracy (NS < 0.65) for predicting ACP and DEH (using STF) (Figs 2 and 4). Overall, the ACP, ALP, and DEH were predicted using the PLSR method with very well accuracy (0.75 ≤ R^2^_val_*<*0.90) and by STFs with good accuracy (0.65 ≤ R^2^_val_*<*0.75).

Visual inspection indicated that the enzyme activity-related spectral information is concentrated in a subset of important wavelengths (Fig 4 and Eqs 1 to 3). In addition, the shared area of notable wavelengths disclosed that nearly all algorithms successfully identified essential reflectance signatures at 1364 nm (related to the C-H combination band of CH_3_ groups), 1896 nm (corresponding to the 2nd overtone of C = O stretching of CH_2_O groups), 2193 nm (associated with amide), and 2337 nm (linked to the 3rd overtone of COO stretching of CH_3_ in proteins) [68]. These identified signatures have been previously associated with being responsible for enzyme activity [69]. Moreover, the bands of 1400 and 1900 nm are related to OH in water molecules and 2250 to 2500 nm are related to methyl [70]. According to Table 2, there were significant correlations between the studies soil enzymes activity and basic physico-chemical attributes, i.e., ACP with clay, pH, and MWD (r values of 0.23, 0.26, and -0.33, respectively); ALP with sand, clay, and ln (EC) (r values of -0.30, 0.54, and -0.45, respectively); and ln (DEH) with clay, pH, MWD, and ln (EC) (with r values of 0.45, -0.30, 0.35, and -0.31, respectively). As these basic soil attributes can be reliably predicted by Vis-NIR spectroscopy, therefore the observed relationships may have helped the good and reasonable predictions of the studied enzymes activity.

Considering the fact that some of the enzymes assayed are based on organic compounds with functional groups that are able to absorb radiation in the NIR region and provoke direct changes in the reflectance characteristics of the samples, the Vis-NIR spectroscopy may show good capability to predict individual enzyme activities [71].

Other studies reported that better results for predicting microbiological parameters like DEH with R^2^ = 0.96 [72]. In addition, PLSR predictive approaches have been applied to successfully assess enzymes activity parameters in NIR and Vis-NIR ranges with R^2^ values of 0.80 and 0.78, respectively [24].

High R^2^ values (> 0.80 and as high as 0.93) have been presented for predicting enzymatic activities by PLSR in several studies [21,71,73,74] which was consistent with our research. However, fair and comparatively poor performances (R^2^< 0.60) was obtained for the prediction of arylsulfatase, DEH, and urease in the research conducted by Mondal et al. [75]; ACP, arylsulfatase, and DEH in the study of Reeves and McCarty [76]; acid phosphatase and arylsulfatase [77].

Comino et al. [71], predicted the activity of enzymes in calcareous soils of Jaén province, Spain with NIR spectroscopy. They reported that a large number of latent variables were necessary, probably due to the most important sources of spectral variability, which are related to soil mineralogy. Their findings showed that the PLSR model by using NIR spectroscopy predicted β-glucosidase and ALP wit acceptable accuracy (cross validation R^2^ of 0.60 and 0.63, respectively); arylsulphatase with moderate accuracy (cross validation R^2^ = 0.46); and ACP and DEH activity with poor accuracy (cross validation R^2^ of 0.33 and 0.23, respectively).

To establish a dependable database for forecasting enzyme functions, it is crucial to focus on the precision and suitability of the analytical methods [24]. It is essential to meticulously optimize assays, taking into account factors such as pH, and also consider the impact of substrate inhibition, binding, non-Michaelis-Menton kinetic enzyme reactions, and the existence of isofunctional enzymes in samples from the environment, as deliberated by German et al. [78].

#### Prediction of SMR, P_mic_, and C_mic_

The PLSR and STFs created utilizing Vis-NIR spectra successfully predicted SMR, P_mic_, and C_mic_ with good accuracies based on NRMSE_val_ and NS_val_ classification guidelines, respectively. In addition, the R^2^_val_ and RPIQ_val_ demonstrated reliable predictions of P_mic_ (using both PLSR and STF) and C_mic_ (using PLSR model); good prediction of SMR (using both PLSR and STF) and C_mic_ (using STF) by Vis-NIR spectra.

The acceptable to very good predictions of SMR, P_mic_, and C_mic_ can be attributed to their significant correlations with the basic soil physico-chemical attributes which can be accurately predicted with Vis-NIR spectroscopy. As shown in Table 2, the SMR is significantly correlated with pH and ln (EC) (r values of -0.32 and 0.26, respectively); P_mic_ with SOM, CCE, sand, clay, and ln (EC) (r values of 0.32, -0.36, -0.26, 0.24, and -0.26, respectively); and C_mic_ with SOM, pH, and ln (EC) (r values of 0.24, -0.46, and 0.25, respectively).

Quantifying microbial biomass can be beneficial for improving the assessment of nutrient cycling in soil [79]. NIR and Vis-NIR exhibited the most accurate predictions for C_mic_, with outcomes categorized as moderately successful (R^2^ values ranging from 0.82 to 0.84) [76]. The substrate-induced respiration, indicating the soil microbial biomass’s capacity to react to added substrate, has been forecasted with moderate accuracy [80] and, in other studies, with good [81] accuracy. In the case of P_mic_, the accurate prediction was reported by Terhoeven-Urselmans et al. [82] using Vis-NIR (R^2^ = 0.81). Gandariasbeitia et al. [81] in calcareous soils of northern Spain, reported Vis-NIR spectroscopy by using PLSR modeling approach is able to accurately predict the SMR (basal respiration, BR, in the addressed reference) by R^2^ ≥ 0.86. Prediction of C_mic_ and P_mic_ was reasonably good, probably due to close relationship between them and SOM (Tabel 2). The organic compounds in the SOM can significantly affect the absorption of radiated Vis-NIR spectra, as found by Zornoza et al. [21] and Comino et al. [71].

In addition, they reported assigned wavelengths for the C_mic_ prediction of field-moist soils were at 1408, 1842, and 2414 nm. Also, important wavelengths for Basal respiration were 1836 and 2274 nm (both alkyl groups) and 1510 nm (amino groups). Our findings align with the results reported by the investigators mentioned (Fig 5 and Eqs 4 to 6).

According to literature, some soil properties including particle-size distribution (PSD), lime (CaCO_3_), "EC", "pH", and "SOM" are recognized to influence soil spectral reflectance data across the entire spectrum [13,16,25,83,84]. Consequently, it is reasonable to anticipate that biological attributes could be reasonably predicted using spectroscopy-based approaches.

### Assessing the efficacy of the created PLSR models and STFs in predicting soil biological attributes

Fig 6 represents data on R^2^_cal_ and R^2^_val_, respectively, comparing PLSR models and STFs developed for predicting selected biological attributes. The PLSR models, in contrast to STFs developed, exhibited improved prediction potentials for all the studied biological attributes using "Vis-NIR" reflectance spectra. The R^2^ values for PLSR models in comparison with STFs showed increases of 10.6, 4.1, 21.2, 7.6, 6.67, and 8.70% for R^2^_cal_ values and 16.18, 11.9, 16.9, 6.58, 6.94, and 6.52% for R^2^_val_ values to predict ACP, ALP, ln (DEH), SMR, P_mic_, and C_mic_, respectively.

In summary, the PLSR model consistently outperformed STFs in predicting all studies biological parameters, albeit without a significant difference. The most notable distinction in predictive capability between the established "PLSR" and "STFs" methods was evident in the prediction of ln (DEH), showcasing an R^2^_cal_ difference of 0.13 (17.2%). All analyzed soil biological properties were predicted with R^2^_val_ and R^2^_cal_ values of ≥ 0.65 using the applied Vis-NIR spectroscopy-based methods of PLSR and STFs.

Our results closely align with the discoveries reported by Mozaffari et al. [13] who concluded that PLSR models exhibited superior predictive capabilities compared to STFs in forecasting the majority of examined soil physical and chemical characteristics within their investigated calcareous soils. However, similar to our results, they reported that there are no significant differences between the capabilities of the aforementioned approaches. STFs proved to be user-friendly and applicable, demonstrating good and very good accuracies in predicting enzymes activities and microbial biomass. This affirms the soundness and appropriateness of our procedure in selecting essential Vis-NIR reflectance bands for STF development [13].

In conclusion, this method involved the strategic selection of effective bands to develop STFs and predict soil biological properties, yielding relatively satisfactory results. Further exploration of this model to predict other soil health indicators, including physico-chemical and biological properties, is recommended. Additionally, the simplicity, practicality, and effectiveness of this model could make it a viable option for studying and predicting other biological attributes.

## Conclusion

Significant correlations were observed between the basic soil properties and the studied biological parameters. Basic soil physical and chemical properties can be accurately predicted by Vis-NIR spectroscopy. Therefore, correlations between them and the target biological properties may increase the predictions accuracy, when Vis-NIR spectroscopy approach is used. Results revealed that the ACP, ALP, ln (DEH), SMR, P_mic_, and C_mic_ attributes were predicted with acceptable to very good accuracies (0.65 ≤ R^2^_val_ ≤ 0.81) using Vis-NIR spectroscopy by applying PLSR and SMLR modeling approaches. Our findings underscore that the developed PLSR models exhibit superior accuracy in predicting the majority of key soil biological attributes compared to STFs. Although the performance differences between the two methods were not significant, it is important to emphasize the high recommendation for utilizing the developed STFs. The STFs were developed using the minimum 7, for predicting C_mic_, and maximum 11, for predicting P_mic_, effective spectral reflectance bands in Vis-NIR region. These STFs prove highly effective in predicting soil enzymes activities and selecting biological factors, specifically those parameters investigated in this study, within calcareous soils for precise soil mapping. Additionally, ongoing enhancement of equations can be achieved by incorporating more data points into the calibration models, linking laboratory-measured values with Vis-NIRS values. It’s worth noting that the equipment required for Vis-NIR spectroscopy is cost-effective, making it a viable choice for swiftly analyzing a vast number of samples in scenarios demanding rapid assessments. It should be noted that although soil is a very complex matrix and accurate estimation of soil enzyme activity by spectrometer method is very difficult, very good and excellent estimation of some basic physical and chemical characteristics and their correlation and strong relationship with soil biological properties (such as soil enzymes) can be provided, which enables us to estimate some soil biological properties using this approach with good accuracy.

## Supporting information

S1 Raw data Raw data is available as supplementary file. (XLSX)
